# Experimental Study on Ion Transport in Microfluidic Electrodialysis Using Partially Masked Ion Exchange Membranes

**DOI:** 10.3390/mi13030356

**Published:** 2022-02-24

**Authors:** Junsu Jang, Minsung Kim, Joonghan Shin, Daejong Yang, Minseok Kim, Bumjoo Kim

**Affiliations:** 1 Department of Future Convergence Engineering, Kongju National University, Cheonan 31080, Korea; vpvpwkrfur@naver.com (J.J.); adrt20206@naver.com (M.K.); jhshin@kongju.ac.kr (J.S.); daejong@kongju.ac.kr (D.Y.); 2 Department of Mechanical and Automotive Engineering, Kongju National University, Cheonan 31080, Korea; 3 Department of Mechanical System Engineering, Kumoh National Institute of Technology, Gumi 39177, Korea; 4 Department of Aeronautics, Mechanical and Electronic Convergence Engineering, Kumoh National Institute of Technology, Gumi 39177, Korea

**Keywords:** ion exchange membrane, electrodialysis, desalination, nanofluidics, electroconvection

## Abstract

Electrodialysis using anion-exchange membranes (AEMs) and cation-exchange membranes (CEMs) has been widely used for water desalination and the management of various ionic species. During commercial electrodialysis, the available area of an ion-exchange membrane is reduced by a non-conductive spacer that is in contact with the AEM/CEM. Although multiple reports have described the advantages or disadvantages of spacers, fewer studies have explored the effects of spacers on the mass transport effect of the reduced membrane area excluding the fluid flow change. In this paper, we present our experimental studies concerning mass transport in microfluidic electrodialysis systems with partially masked ion-exchange membranes. Six different types of masking membranes were prepared by the deposition of non-conductive films on parts of the membranes. The experimental results showed that the overlapped types (in which masking was vertically aligned in the AEM/CEM) exhibited a larger electrical conductance and better current/energy efficiency, compared with the non-overlapped types (in which masking was vertically dislocated in the AEM/CEM). We also observed that a reduction in the unit length of the unmasked ion-exchange membrane enhanced overall mass transport. Our results demonstrate the effects of patterned membranes on electrical resistance and desalination performance; they also identify appropriate arrangements for electromembrane systems.

## 1. Introduction

Ion-exchange membranes featuring many nanopores have attracted considerable attention from researchers in various fields; such membranes exhibit unique mass transport characteristics and ion selectivity. Workers in the field of eco-friendly and renewable energy commonly use ion-exchange membranes during the analysis of fuel cells, redox flow batteries, electrodialysis, and reverse electrodialysis [1,2,3,4]. Electrodialysis is a mature technology; desalination employs ion-exchange membranes [2,3,4]. Electrodialysis (which is driven by electrical energy) is simple, scalable, and easily controllable; it efficiently treats brackish water [5]. A conventional electrodialysis system features ion-exchange membranes, electrodes, a spacer, and fluidic compartments; it operates via the electrophoretic migration of cationic/anionic species (driven by an electric field) through permselective membranes [2]. A spacer embedded between the anion-exchange membrane (AEM) and the cation-exchange membrane (CEM) is typically used to separate the membranes, thus providing a physical space for fluid flow [6,7,8]. The spacer (if meshed) also promotes electrolyte mixing in the fluidic channel [9]. Woven or non-woven meshes fabricated from non-conductive polymer materials are commonly used; the winding and complicated flow becomes turbulent, enhancing bulk electrolyte mixing [8,10,11,12]. Such mixing (caused by geometrical features) mitigates concentration polarization near the membranes, thus reducing the electrical resistance of the fluidic channel [6,7,9]. Considering these advantages, several experimental and numerical studies have sought to modify the geometry of the net to change the flow patterns and further alleviate concentration polarization [9,13,14,15,16,17]. Studies regarding multi-layer spacers (i.e., a middle spacer and two thin outside spacers) revealed that such spacers exhibited Sherwood numbers that were 30% higher (at the same crossflow power consumption) than the Sherwood numbers of commercial, non-woven single-meshed spacers [11,18]. Although the spacer mitigates concentration polarization (as described above), the intrinsic limitation of the shadow effect (also termed the screening effect; i.e., the reduction in membrane area involved in ion exchange) has been a considerable problem [19,20]. Alternative approaches include the use of an ion-exchange resin or a profiled membrane without a spacer [20,21,22,23,24,25,26,27]. A spacer coated with ion-exchange resin performed better as compared to a non-coated spacer because ion conductivity was enhanced [21]. Another study presented a novel ion-exchange membrane that protruded from the membrane surface; the membrane exhibited lower hydraulic friction (and hence, a higher Reynolds number) than did a conventional spacer stack [22,23,24,25,26,27].

Although many efforts have been made to overcome the shadow effect and improve the electrical performance in electrodialysis, few experimental studies concerning the shadow effect itself (not the coupling results after chaotic flow changes in meshed structures) have been performed. It is impossible to decouple the effects of ion-exchange membrane screening when a spacer embeds between the AEM and CEM. When performing fundamental studies of partially masked ion-exchange membranes (i.e., experiments decoupled from changes in physical flow), it is essential to use a microscale electrodialysis device with partially masked ion-exchange membranes and no spacer between the AEM and the CEM.

In this study, we examined the electrical responses of a microfluidic electrodialysis system with partially masked ion-exchange membranes. We prepared six microscale electrodialysis devices in which the AEM/CEM masked lengths, locations, and vertical overlap ratios differed. The current–voltage responses associated with the various types of masking are presented. In addition, we conducted a comparison of the desalination performance in terms of current efficiency and the energy required for ion removal. We visualized the fluidic channels to observe the flow behaviors on the AEM/CEM masked surfaces; we added fluorescent dye to the feed solution. Our results should aid in electromembrane-mediated desalination.

## 2. Materials and Methods

### 2.1. Concept

**Figure 1:**
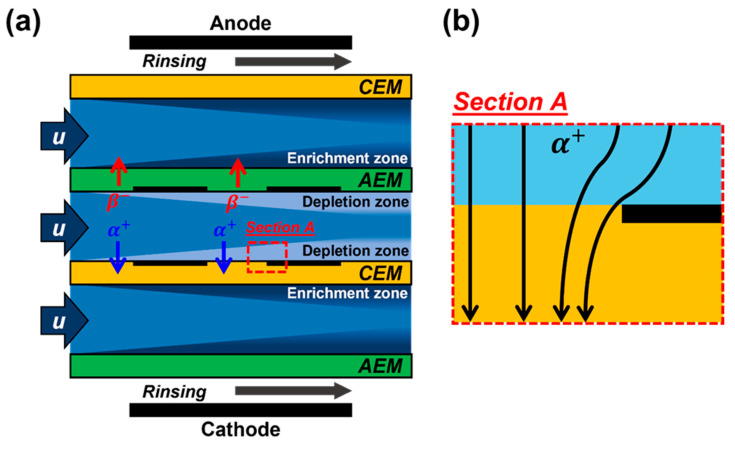
A schematic of electro-membrane desalination and the ionic current path. (**a**) Electrodialysis and (**b**) the ionic current path when an ion-exchange membrane is partially masked. Blue and red arrows indicate the transport of cations (alpha) and anions (beta) by the electric field. The less bright region shows the ion depletion zone and the darkest region shows the ion enrichment zone. An anion-exchange membrane (AEM) and a cation-exchange membrane (CEM) are used for counter-ion transport. Section A (red dotted box in the depletion zone near the CEM) is magnified on the right; it shows the modified cation path.

Electrodialysis features two types of ion-exchange membranes. Depending on the surface charge polarity, a CEM allows only cations to pass; an AEM allows only anions to pass. Figure 1a shows a schematic of electrodialysis; the cations/anions migrate toward the cathodic and anodic sides of the electric field, respectively. Considering the ion-selective membrane properties, local ion-depleted and -enriched streams are generated, thus triggering ion concentration polarization across the ion-exchange membranes. Because of the opposite polarities of the charged surface, ion depletion zones develop on the CEM surface of the anodic side and on the AEM surface of the cathodic side. Therefore, as flow develops, the salt concentration (i.e., the salinity) of the main channel (the second channel from the top) is diluted by the two depletion streams, whereas the salt concentrations of the side channels (the first and third channels from the top) become more concentrated because they host the two enrichment streams. This is the basic principle of electrodialysis.

During conventional electrodialysis, a spacer is embedded between the ion-exchange membranes to accommodate fluid flow in a fluidic channel, although this partially shields the membrane surfaces if the spacer is non-conductive. As shown in Figure 1b, such local membrane screening creates several problems related to the so-called “shadow effect”, including non-uniform ionic current flux and decreases in the effective membrane areas. However, it is difficult to evaluate the effect of partial membrane masking on ion transport in the absence of the spacer because the flow pattern is changed by the spacer. Thus, we fabricated microfluidic channels containing masked ion-exchange membranes without spacers.

### 2.2. Fabrication and Experimental Setup

**Figure 2:**
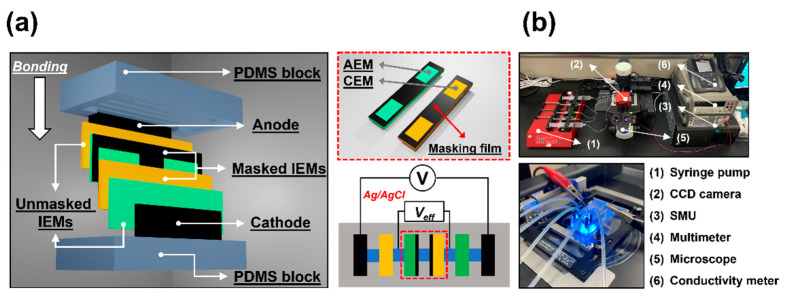
(**a**) Schematics of PDMS-based microscale electrodialysis fabrication and the masked ion-exchange membranes. Masking film of 30 µm thickness was deposited onto the ion-exchange membranes (a pair of AEM/CEM) to physically block the nanopores. The masked membranes were embedded in the second channel (the main channel) from the top; the unmasked membranes were embedded in the first and third channels. The Ag/AgCl electrode was inserted between the first and second channels to measure the electrical responses in the main channel (red dotted box at bottom). (**b**) A photograph of the overall experimental setup.

Figure 2a shows a schematic of the fabrication of polydimethylsiloxane (PDMS)-based microscale electrodialysis systems with partially masked ion-exchange membranes. To ensure that commercial ion-exchange membranes and electrodes of high aspect ratio (i.e., with deep slots) could be placed between shallow microfluidic channels, the PDMS mold was fabricated using a three-dimensional printer (SLA ProJet 7000 HD, 3D systems, Rock Hill, PA, USA) that readily creates microstructures of high aspect ratio [5,28,29,30,31,32,33]. The two PDMS blocks featured four and two pairs of deep slots for the membranes and electrodes, respectively. After the membranes (AMHPP/CMHPP; MEGA Inc., Hodonín, Czech Republic, USD 173/m^2^) and electrodes (Spectracarb 2050A-1550; Fuel Cell Store, College Station, TX, USA) were added, the bottom PDMS block was bonded immediately after plasma treatment (Cute; Femto Science, Hwaseong, Korea).

We modified two ion-exchange membranes (one CEM/one AEM); four and two unmasked ion-exchange membranes were used in their original states. The non-conductive layers on the ion-exchange membrane were created by using non-conductive masking film (TP-1031BSM; Nitto Denko, Osaka, Japan) of 30 µm thickness. Note that the partially masked membranes were placed in front of the main channel (i.e., in the dilute channel, the second channel from the top) to ensure that they dramatically affected the ionic current flux; the electrical resistance of electrodialysis is dominated by the depletion zone.

Figure 2b shows the experimental setup for microfluidic electrodialysis; this allows visualization of flow/concentration and measurement of electrical responses. To apply electric potential and measure the current between the electrodes (i.e., from the anode to the cathode), we used a source measurement unit (SMU, Keithley 236; Keithley Instruments Inc., Cleveland, OH, USA) when shear flow (1 mm/s) was applied by a syringe pump (Fusion 200-X; Chemyx Inc., Stafford, TX, USA). To measure the specific potential of the dilute channel, Ag/AgCl electrodes were connected to a multimeter (34401A; Agilent Technologies, Inc., Santa Clara, CA, USA). We used a fluorescent dye (Alexa 488 triethylammonium; Thermo Fisher Scientific, Waltham, MA, USA) as a salt concentration tracer when visualizing flow and mass transfer. In addition, a benchtop conductivity meter (Star A215 pH/conductivity meter; Orion/Thermo Fisher Scientific) was used to monitor concentration changes in the dilute channel; flow-thru conductivity probes (16-900 flow-thru conductivity electrode, Microelectrodes Inc., Bedford, NH, USA) were placed downstream. Sodium chloride solution (NaCl, 10 mM) served as the feed in all experiments while sodium sulfate solution (Na_2_SO_4_, 5 mM) was used as rinsing solution.

### 2.3. Ion-Exchange Membranes with Non-Conductive Masking Films

**Figure 3:**
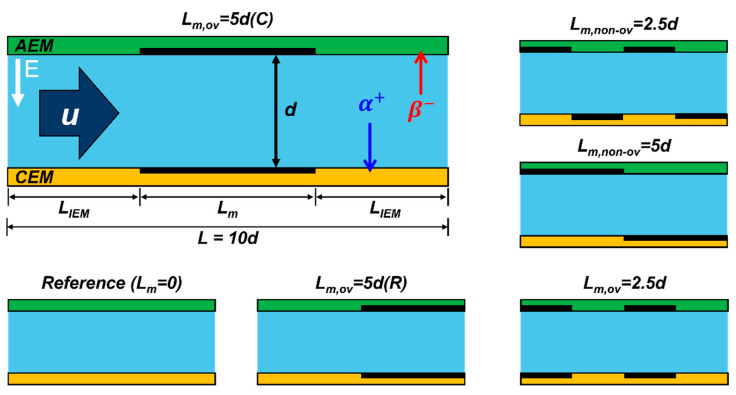
Schematic of the main channel with the five types of masking. All membranes were prepared using non-conductive masking film to cover the same areas, but the patterns differed in terms of unit masking length and local position. “d” is the intermembrane distance between the AEM and the CEM, and thus, represents the width of the fluidic flow channel. “L_IEM_” and “L_m_” are the unmasked and masked lengths, respectively. The total length, “L” (= 10d = 15 mm), is the sum of the masked and unmasked lengths. L_m,ov_ and L_m,non-ov_ differed according to the vertical alignment of the masked regions of the AEM/CEM. Of the L_m_ = 5d membranes, the masking film was located on the right side (L_m,ov_ = 5d (R)) or the center of the membrane (L_m,ov_ = 5d (C)).

To investigate the effects of the masking pattern and masking itself on the ion-exchange membranes, we prepared five different types of AEM/CEM membranes that differed in terms of masked length, position, and vertical overlap. Figure 3 shows a schematic of all five types. The intermembrane distance between the AEM and CEM is represented as “d”; this was kept constant at 1.5 mm. The masked and unmasked lengths of the membranes are denoted as “L_m_” and “L_IEM_”, respectively; their sum is the total channel length (L), which, in this study, was held constant at 10 × d (i.e., 10 d) = 15 mm. Most ion transport occurred through the unmasked surfaces of the membranes (L_IEM_), not through the masked regions (L_m_); the non-conductive film totally blocked the nanopores. To ensure a fair comparison of the different masked lengths (L_m_ = 5d, 2.5d), we held the effective (unmasked) membrane length (5d, the summation of L_IEM_) constant at 50% of the total membrane length. We also considered the vertical masking alignment; masking overlap on the AEM and CEM can enhance or hinder ion transport, depending on the configuration. Thus, we distinguished whether the masking positions of the AEM/CEM were the same or different using the descriptors “L_m,ov_” (overlapped) and “L_m,non-ov_” (non-overlapped), respectively. For L_m_ = 5d, we defined two different types of masking depending on the positions of the masking film; L_m,ov_ = 5d (C) and L_m,ov_ = 5d (R) indicated that the film was in the center or on the right side of the membrane, respectively. A reference (no masking film, L_m_ = 0) was also prepared.

## 3. Results and Discussion

### 3.1. Fluorescent Visualization of the Flow Channel

**Figure 4:**
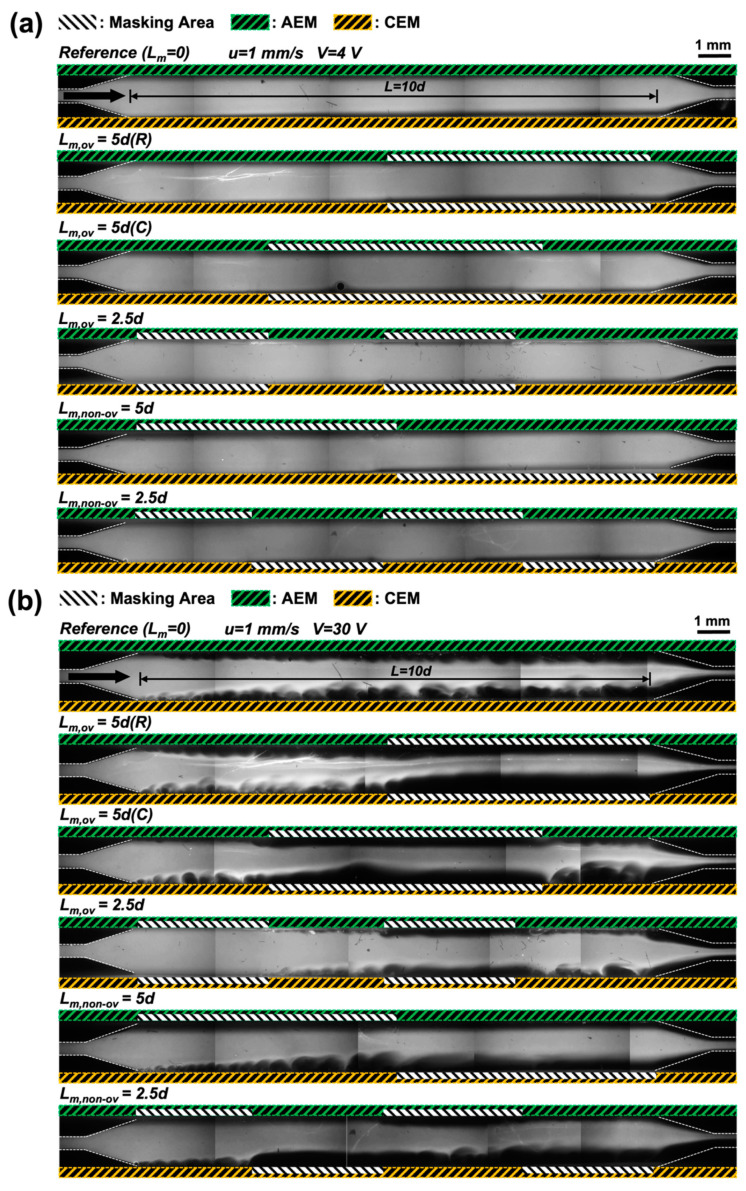
Fluorescence images of the main channel (i.e., dilute channel) at a constant voltage (**a**) V = 4 V (top, limiting regime) and (**b**) 30 V (bottom, overlimiting regime)) under shear flow velocity (u = 1 mm/s) for the six different membranes. Electroconvective vortices were created to explore whether the membranes were adequately masked by the non-conductive film. Vortices developed on the membrane surfaces (green and yellow dotted boxes) but not in the masked areas (white dotted boxes).

After device fabrication, we visualized the main dilute channels of the six different membrane types. We used a fluorescence microscope and NaCl solution with a fluorescence dye to observe the behavior of the ion-depletion zone in each dilute channel. Figure 4 shows the fluorescent visualization of the six different membrane types under the same shear flow velocity (1 mm/s). The yellow and green regions indicate the unmasked AEM and CEM regions, respectively; the black regions are the masked regions. As shown in Figure 4a, it was difficult to distinguish existence and block of ionic current flow through unmasked and masked IEMs, respectively, due to the stable laminar stream at relatively low voltage (4 V, Ohmic-limiting regime). Accordingly, a comparatively high voltage (30 V, overlimiting regime) was applied in all cases to observe electroconvection; it is convenient to assess membrane masking and ion transport by creating electroconvective vortices, such as that shown in Figure 4b. Specifically, vortices arise on membrane surfaces because strong ionic currents pass through the membranes at the overlimiting regime. On the other hand, growing vortices are not observed on the masked surfaces (non-conductive film deposited) of ion-exchange membranes; the vortices develop near the membranes. In general, vortices no longer grow when the feed flow enters masked membrane regions; vortices grow again when they meet the unmasked membrane regions. We thus confirmed that the masking film prevented ion transport through the membranes by physically blocking the nanopores.

### 3.2. Current Density–Voltage Response

**Figure 5:**
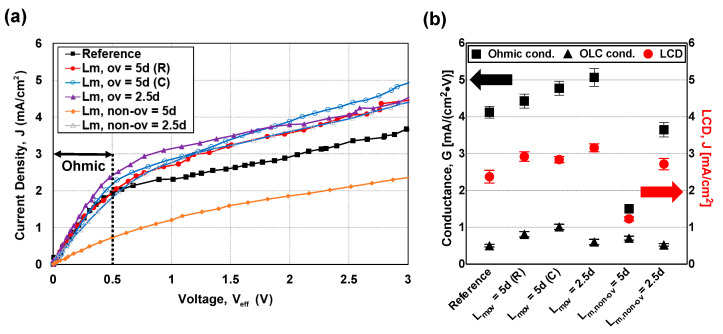
Electrical responses of the six different types as revealed by (**a**) current density–voltage curves. The current was measured between the anode and the cathode at a voltage sweep rate of 0.2 V/30 s, from 0 to 3 V. The voltage (V_eff_) was measured by Ag/AgCl electrodes; this was the cell voltage of the main (dilute) channel. (**b**) Ohmic conductances and limiting current densities (LCD). The Ohmic conductances were calculated from the experimental data (the J–V curve) of the Ohmic (V_eff_ < 0.5 V) regime at a limiting current density of V_eff_ = 0.5 V. All experiments were repeated at least three times; the results were similar. The Ohmic conductances indicated that the mass transports of L_m,ov_ membranes were higher than the mass transports of the other patterned (L_m,non-ov_) and reference membranes.

Figure 5a shows the electrical responses of the system for the various membrane types (reference, L_m,ov_ = 5d (R), L_m,ov_ = 5d (C), L_m,ov_ = 2.5d, L_m,non-ov_ = 5d, and L_m,non-ov_ = 2.5d). We measured the current between the anode and the cathode at a voltage sweep rate of 0.2 V/30 s and a constant shear flow velocity (1 mm/s). To monitor only the voltage drop of the main channel (thus excluding the voltage drops of the anodic/cathodic rinsing channels), we installed Ag/AgCl electrodes that measured only the effective voltage (V_eff_). As shown in the current density and voltage curves, the Ohmic (V_eff_ < 0.5 V) and overlimiting (V_eff_ > 0.5 V) regimes were readily distinguishable in all cases; the Ohmic conductances and limiting current densities are summarized in Figure 5b.

The electrical responses demonstrated two clear tendencies. First, the Ohmic conductance of the L_m,non-ov_ = 5d membrane was considerably lower than the Ohmic conductances of the other membranes. Dislocation of the AEM/CEM masked regions apparently hindered uniform ion transport and, thus, increased the electrical resistance of the main channel. Notably, dislocation of the masked regions did not always greatly increase the electrical resistance; the Ohmic conductance of the L_m,non-ov_ = 2.5d membrane remained higher than the Ohmic conductance of the L_m,non-ov_ = 5d membrane and was similar to the Ohmic conductance of the other overlap types. Therefore, pattern dislocation itself does not dominate Ohmic conductance, although it can be important when the masked length is long. It is reasonable to consider the masked length relative to the intermembrane distance; ion transport is determined by both the horizontal and vertical length. Second, a short unmasked length (L_IEM_) enhanced mass transport; the Ohmic conductance rose in the following order: reference (L_m_ = 0), L_m,ov_ = 5d (R), L_m,ov_ = 5d (C), and L_m,ov_ = 2.5d. It was previously reported that a longer the ion-exchange membrane leads to higher electrical resistance and lower areal efficiency, considering the increasing thickness of the ion-depletion zone [5]. Thus, to enhance ionic current through an ion-exchange membrane, the unit membrane length exposed to the electrolyte should be short because the total membrane length is constant. In our experiments, the unit unmasked length (L_IEM_) values of the reference, L_m,ov_ = 5d (R), L_m,ov_ = 5d (C), and L_m,ov_ = 2.5d membranes were 10d, 5d, 2.5d, and 2.5d, respectively. Thus, the trend that we observed is consistent with the cited report. We also provide overlimiting conductance for all types, but their values are relatively small and do not show significant differences.

### 3.3. Desalination Performance

**Figure 6:**
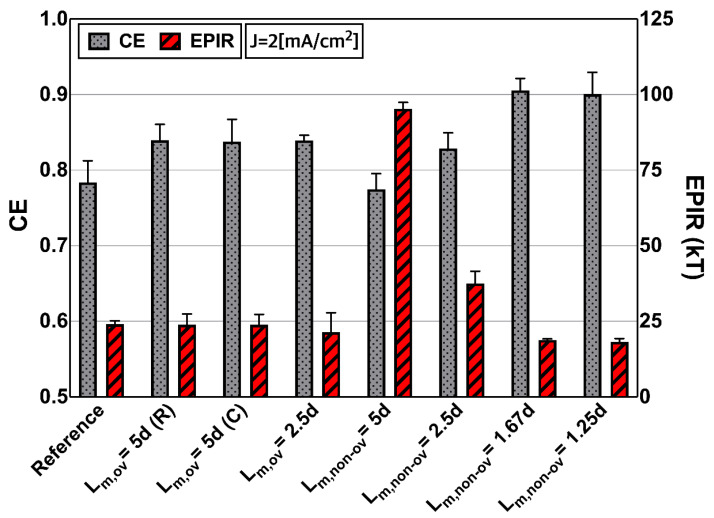
Desalination performances afforded by partially masked ion-exchange membranes. The CE and EPIR values were calculated from desalination experiments performed at constant current density (J = 2 mA/cm^2^). The CE values of L_m,ov_ membranes were enhanced to a greater extent than the CE values of the other membranes (L_m,non-ov_, reference). In terms of the EPIR values (expressed as functions of CE and voltage (V_eff_)), the values for the non-overlapped membranes were almost twofold greater than the values for the L_m,ov_ and reference types, principally because the EPIR values are dominated by voltage increments rather than the CE values.

We next examined the desalination performances of the various masked membranes. Figure 6 shows the experimental current efficiencies (CEs) and energy per ion removal (EPIR) at a constant current density (J = 2 mA/cm^2^) for all masked patterns. We now introduce CE and EPIR as follows:

(1) $$\mathrm{CE}=\frac{{\mathrm{zFQ}}_{\mathrm{desalted}}{(C}_{0}{-C}_{\mathrm{desalted}})}{\mathrm{NI}}$$

(2) $$\mathrm{EPIR}=\frac{{\mathrm{IV}}_{\mathrm{eff}}{/Q}_{\mathrm{desalted}}}{{\mathrm{zk}}_{B}{T(C}_{0}{-C}_{\mathrm{desalted}})}\;\propto \;\frac{V_{\mathrm{eff}}}{\mathrm{CE}}$$

where z, F, k_B,_ and T indicate the ion valence, Faraday’s constant (96,500 C·mol^−1^), Boltzmann constant and temperature, respectively. V_eff_ is the effective voltage, I is the current, N (=1) is the electrodialysis cell number, and C_0_ is the feed bulk concentration. C_desalted_ and Q_desalted_ are the concentration and volumetric flow rate of the desalted channel (i.e., the main channel), respectively. CE indicates how efficiently current is used for the ion’s removal from desalted streams, and the EPIR is the amount of energy consumed when rejecting the unit ion. Ideally, CE = 1, because all electrical current should reflect counter-ion transport through the ion-exchange membranes. However, the CE becomes < 1 in practical use because of current leakage, the imperfect permselectivities of the CEM/AEM, and back-diffusion [34].

At the same current density (J = 2 mA/cm^2^), the feed concentration (10mM) was desalted to dilute the output with concentration ranging from 9.06 to 9.2mM, and we observed that most partially masked membranes (except the L_m,non-ov_ = 5d membrane) exhibited higher CE values compared with the reference membrane (L_m_ = 0). Thus, although the area of the ion-exchange membrane (L_IEM_) was reduced by the masking film, the current efficiency improved, consistent with the current–voltage responses (Section 3.2). This is principally because a short membrane length (L_IEM_) is associated with less electrical resistance, thereby enhancing mass transport. Furthermore, we found that the non-overlapped membranes (L_m,non-ov_ = 5d) exhibited higher EPIR values (red bars) than did the overlapped types (L_m,ov_ = 5d). As shown above, the non-overlapped membranes were associated with larger voltages and increased electrical resistance in the fluidic channel, eventually increasing the EPIR, which is known to be dominated by the effective voltage (V_eff_). It is assumed that the masking pattern was probably longer relative to the intermembrane distance since the L_m,non-ov_ = 2.5d membrane exhibited a better EPIR than did the L_m,non-ov_ = 5d membrane, although the L_m,non-ov_ = 2.5d membrane was a non-overlapped membrane. As discussed in Section 3.2, because the L_m,non-ov_ = 2.5d is a lower ratio (L_m_/d = 2.5) than L_m,non-ov_ = 5d (L_m_/d = 5), the ion transport of the non-overlapped membrane was not significantly compromised. Based on the assumption, we added two cases (L_m,non-ov_ = 1.67d, 1.25d) to determine critical L_m_ values at which this overlap effect became negligible. As can be seen Figure 6, it is reasonable to assume that L_m,non-ov_ = 2d would be the critical masking length for the overlap effect.

## 4. Conclusions

We experimentally explored the effects of partially masked ion-exchange membranes on the electrical responses and current/energy efficiency of electrodialysis. Non-conductive masking film was patterned onto membranes with or without vertical alignment (overlapped or non-overlapped) of the AEM/CEM. We found that the overlapped membranes exhibited larger Ohmic conductances, compared with the non-overlapped and reference types. In particular, the L_m,ov_ = 2.5d membrane exhibited the largest conductance because its masking and unmasking unit lengths were smaller than the masking and unmasking unit lengths of the other types; this enhanced mass transport through an AEM/CEM. Similar to the Ohmic conductance findings, the limiting current density of the L_m,ov_ = 2.5d membrane type was also large (70% greater than the reference value). In contrast, the non-overlapped membranes exhibited lower Ohmic conductances compared with the overlapped and reference types. In terms of current/energy efficiency, the overlapped membranes exhibited larger current efficiencies and better EPIRs than did the other types, considering the enhanced ion transport through the AEM/CEM (as mentioned above). Thus, our results suggest that shortening of the masking length and overlapping of the masking pattern improved mass transport, as compared with transport in the absence of masking (i.e., the reference type). Regarding the masking length, although it was a little bit larger than those in the actual electrodialysis (~0.1 mm) and a previous numerical study with smaller pattern size has been reported [35], this is a first experimental demonstration of partially masked ion-exchange membranes, and follow-up studies with more exquisite screening patterns can be expected. Based on the scientific studies on microfluidic systems, it is also meaningful to apply real industrial electrodialysis system. Therefore, we believe that our work will serve as the basis of future studies exploring geometrical factors that further enhance electromembrane performance.

## Data Availability

Not applicable.
